# Supplementary figures and images for: Quantifying influence of human choice on the automated detection of Drosophila behavior by a supervised machine learning algorithm
Source: PLoS One. 2020 Dec 16;15(12):e0241696. doi: 10.1371/journal.pone.0241696 (PMC7743940; doi:10.1371/journal.pone.0241696)

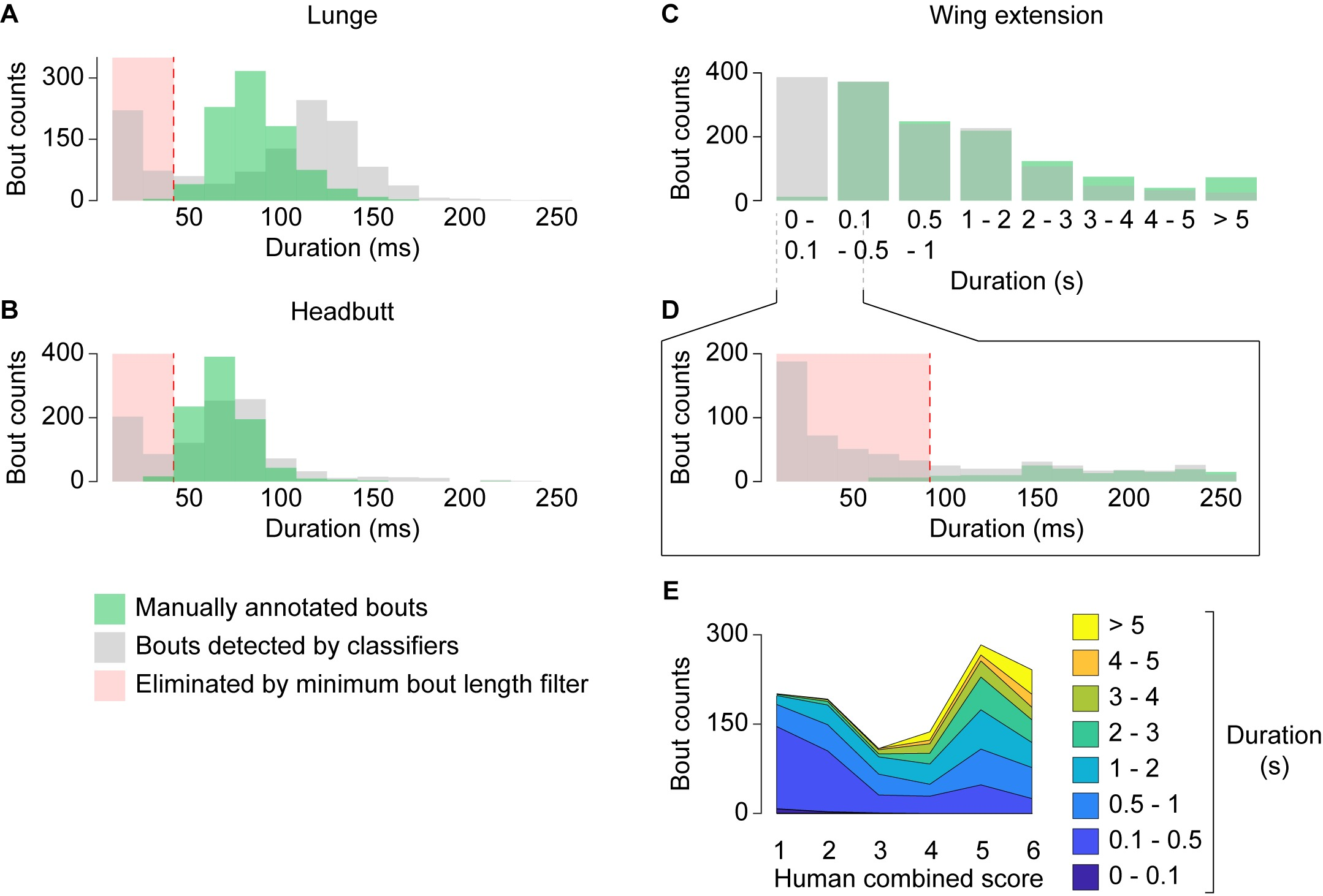

Supplement: S1 Fig — (A-D). Histograms of bout duration for lunge (A), headbutt (B), and wing extension (C, D). Green is the distribution of human annotated bouts, and gray is the distribution of JAABA bouts. For lunges and headbutts, bout duration was binned for every 17 ms (duration of one frame). For (C), bins are indicated below the plot. (D) is the magnified histogram for durations between 0 and 250 ms. Red shades show the durations that are eliminated by the minimum bout length filter (see text and S3 Fig). (E). Distribution of wing extension bout durations according to human combined score. Bins are color-coded as shown on the right. (TIF) [file pone.0241696.s001.tif]

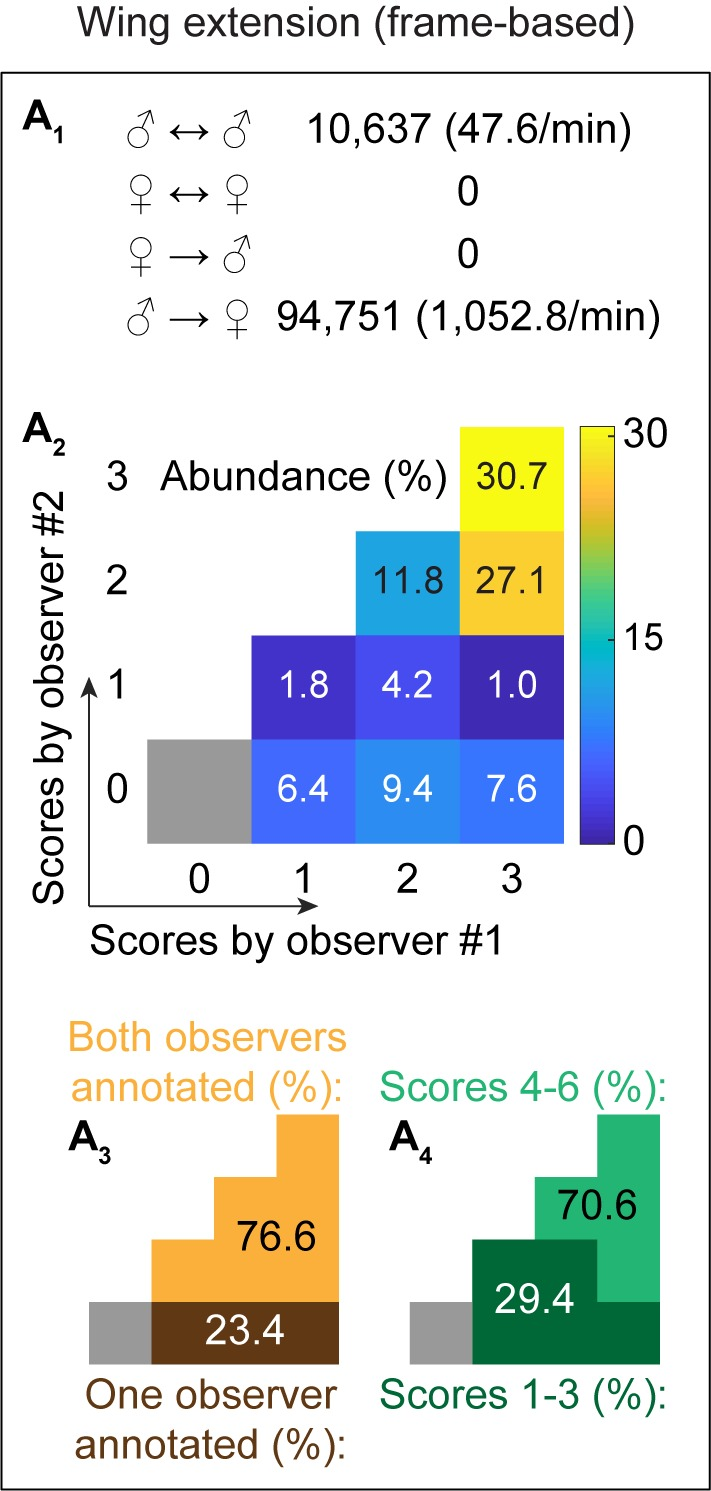

Supplement: S2 Fig — Frame-based summary of human annotations for wing extension (A1), categorized according to interaction types. Distribution of human score combinations are shown in 4-by-4 grid with pseudocolor (A2) that represent relative abundance (scale bars on the right of the grid), and the breakdown according to whether frames were counted by one or two observers (A3) and combined scores (A4) are also shown. Source data is identical to that used in Fig 1B. (TIF) [file pone.0241696.s002.tif]

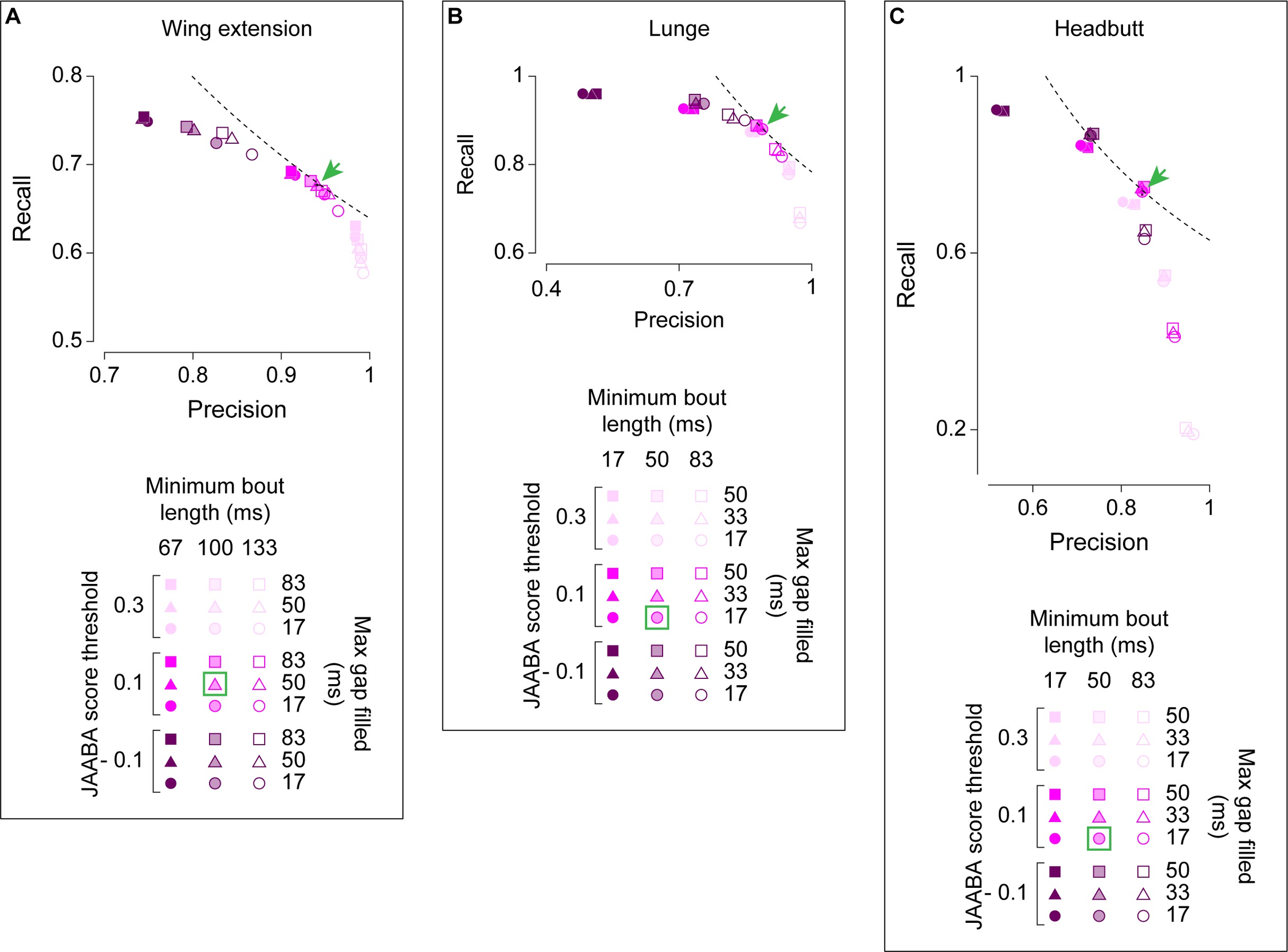

Supplement: S3 Fig — Recall-precision plot of fully trained classifiers for wing extension (A), lunge (B), and headbutt (C) when average JAABA score threshold, minimum bout length, and maximum gap to be filled are varied as indicated below each plot. Green (arrows and rectangles) indicates the parameter combinations chosen for fully annotated classifiers. Dotted lines are inverse proportion functions that pass the points indicated by green arrows. These combinations have the near-maximum recall X precision values for each behavior. (TIF) [file pone.0241696.s003.tif]

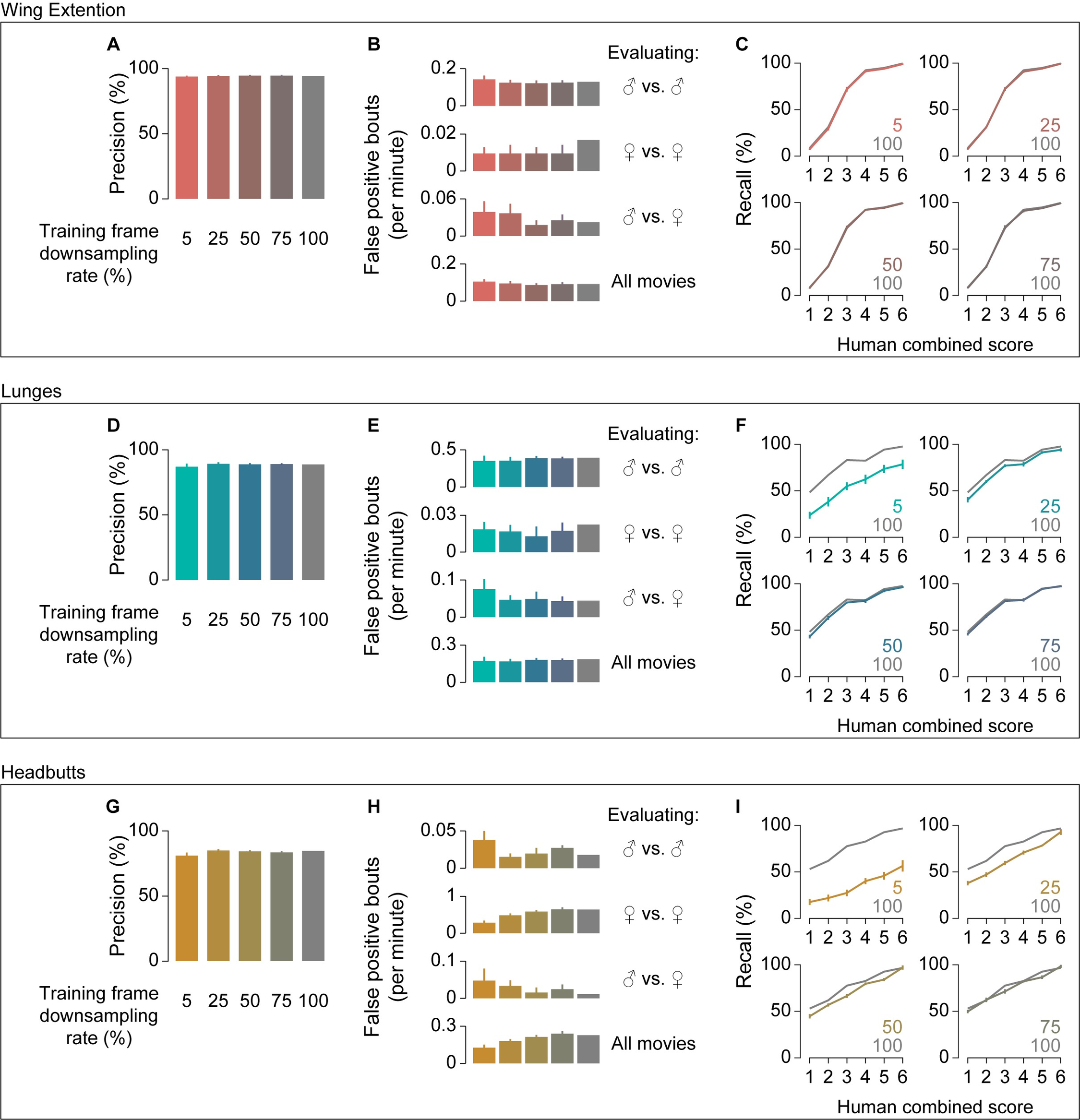

Supplement: S4 Fig — Mean and 95% confidence intervals (vertical lines) of precision (A, D, G), false positive rates (B, E, H) and recall (C, F, I) of classifiers for wing extension (A-C), lunges (D-F), and headbutt (G-I) trained with downsampled frames (indicated below precision plots and inside recall plots). False positive rates are shown separately for types of movies classified as indicated on the right. Values for classifiers trained at 100% downsampling rate (all frames used, shown in gray) are replots from Fig 3. (TIF) [file pone.0241696.s004.tif]

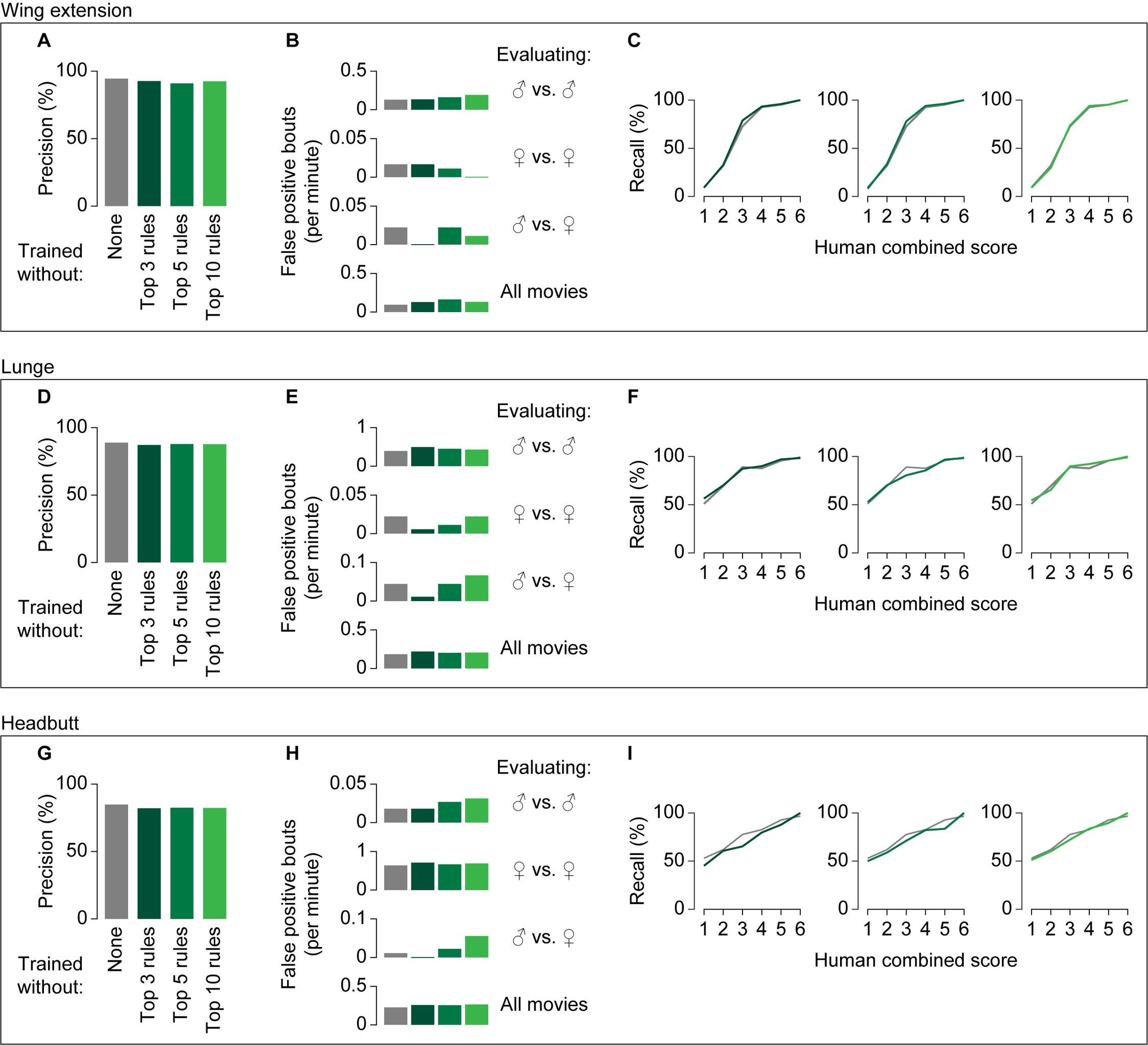

Supplement: S5 Fig — Precision (A, D, G), false positive rates (B, E, H) and recall (C, F, I) of classifiers for wing extension (A-C), lunges (D-F), and headbutt (G-I) trained without the most weighted JAABA rules (indicated below precision plots). False positive rates are shown separately for classifying movie types as indicated on the right. Values for classifiers trained with all rules (shown in gray) are replots from Fig 3. (TIF) [file pone.0241696.s005.tif]

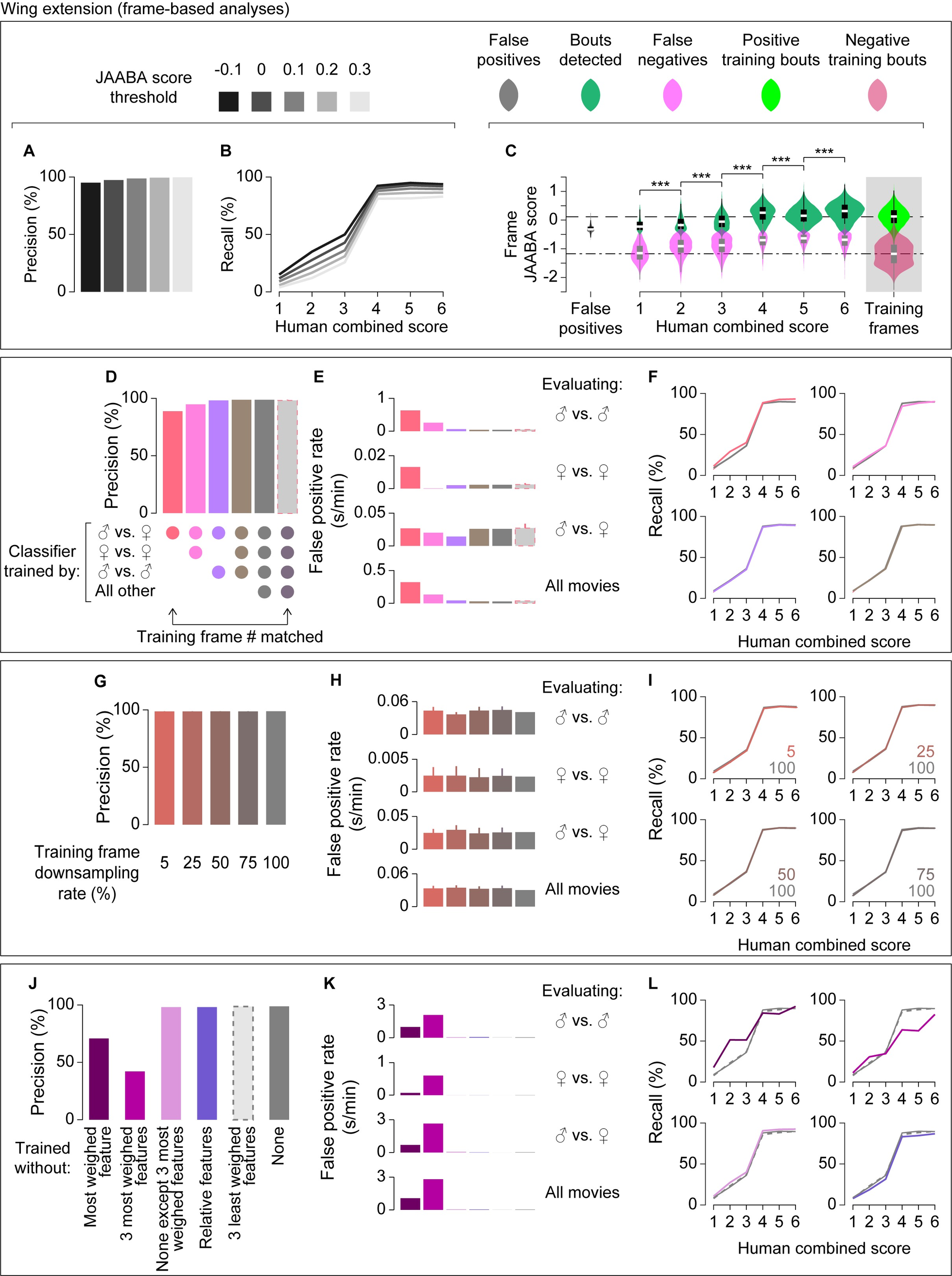

Supplement: S6 Fig — (A-C) Frame-based plots for precision (A), recall (B), and average JAABA score distribution for each human combined score (C) of the results from the fully trained wing extension classifier (corresponding to Fig 3A–3C). (D-F) Frame-based plots for precision (D), false positive rates (E), and recall (F) of the results from the wing extension classifiers trained with the specific types of training movies (shown below precision plots) (corresponding to Fig 4A–4C). (G-I) Frame-based plots for mean and 95% confidence intervals (vertical lines) of precision (G), false positive rates (H) and recall (I) of classifiers trained with downsampled frames (rates indicated below precision plots and inside recall plots). False positive rates are shown separately for classifying movie types as indicated on the right. Frame-based plots for precision (J), false positive rates (K), and recall (L) of the results from the wing extension classifiers trained with subsets of features (as indicated below precision plots). (corresponding to Fig 5A–5C). For (D-L), dark gray plots represent the value of the fully trained classifier (bars in (D), (G), (J) are replots of (A), and lines in (F), (I), (L) are replots of (B), respectively, at JAABA score threshold of 0.1). (TIF) [file pone.0241696.s006.tif]
